# Poster Session II - A265 ARTIFICIAL INTELLIGENCE IN IBD: CURRENT EVIDENCE AND EMERGING APPLICATIONS

**DOI:** 10.1093/jcag/gwaf042.265

**Published:** 2026-02-13

**Authors:** C Galts, A Wen

**Affiliations:** Gastroenterology, McMaster University, Hamilton, ON, Canada; Gastroenterology, McMaster University, Hamilton, ON, Canada

## Abstract

**Background:**

The increased use of Artificial Intelligence (AI) is impacting health care delivery across disciplines, including for patients with Inflammatory Bowel Disease (IBD). Deep learning and machine learning (especially CNNs and radiomics) can process endoscopic, histologic, imaging, and clinical data beyond human capacity. Given IBD’s complexity and dependence on multimodal evaluation, AI is well positioned to impact care in IBD.

**Aims:**

We conducted a narrative review to synthesize and critically evaluate current evidence surrounding AI in IBD across endoscopy, histology, imaging, and clinical applications, highlighting key opportunities and limitations.

**Methods:**

This review combined systematic and narrative approaches. A systematic approach was applied to established domains (endoscopy, histology, imaging) using PubMed, Embase, and Google Scholar (2010 through September 2025). A narrative approach covered emerging applications (digital biomarkers, clinical trials, drug discovery). Data extraction captured AI model type, validation, and diagnostic or predictive accuracy.

**Results:**

A total of 72 studies were included in our review. Substantial evidence demonstrates AI’s efficacy in improving diagnostics and monitoring across endoscopy, histology, and imaging in IBD. Specifically, AI systems using CNNs in endoscopy achieved >90% accuracy for identifying IBD and grading severity, while reproducing indices (Mayo, UCEIS) with high agreement (κ values >0.8 and AUCs up to 0.97). In histology, CNN models achieved >90% accuracy for predicting histologic remission, and models for predicting postoperative CD recurrence achieved exceptional AUCs of 0.98 − 0.99. For imaging, radiomics and CNNs applied to CT, MRI, and IUS achieved AUCs of 0.8 − 0.97 for differentiating IBD types and predicting surgical risk, with IUS models detecting mucosal healing with an accuracy above 90%. AI tools can use biomarkers predicted hospitalization, surgery, and biologic response with AUCs of 0.7 − 0.9. Furthermore, AI reanalysis of UC trial videos improved sensitivity and reduced the necessary sample size by 50%, showing its expanding role in clinical trials and therapeutic advancement.

**Conclusions:**

AI has robust applications in endoscopy, histology, and imaging, and expanding roles in personalized therapy and trial design. Challenges remain in data sharing, standardization, and external validation, but AI is likely to increasingly contribute to the care of IBD patients.

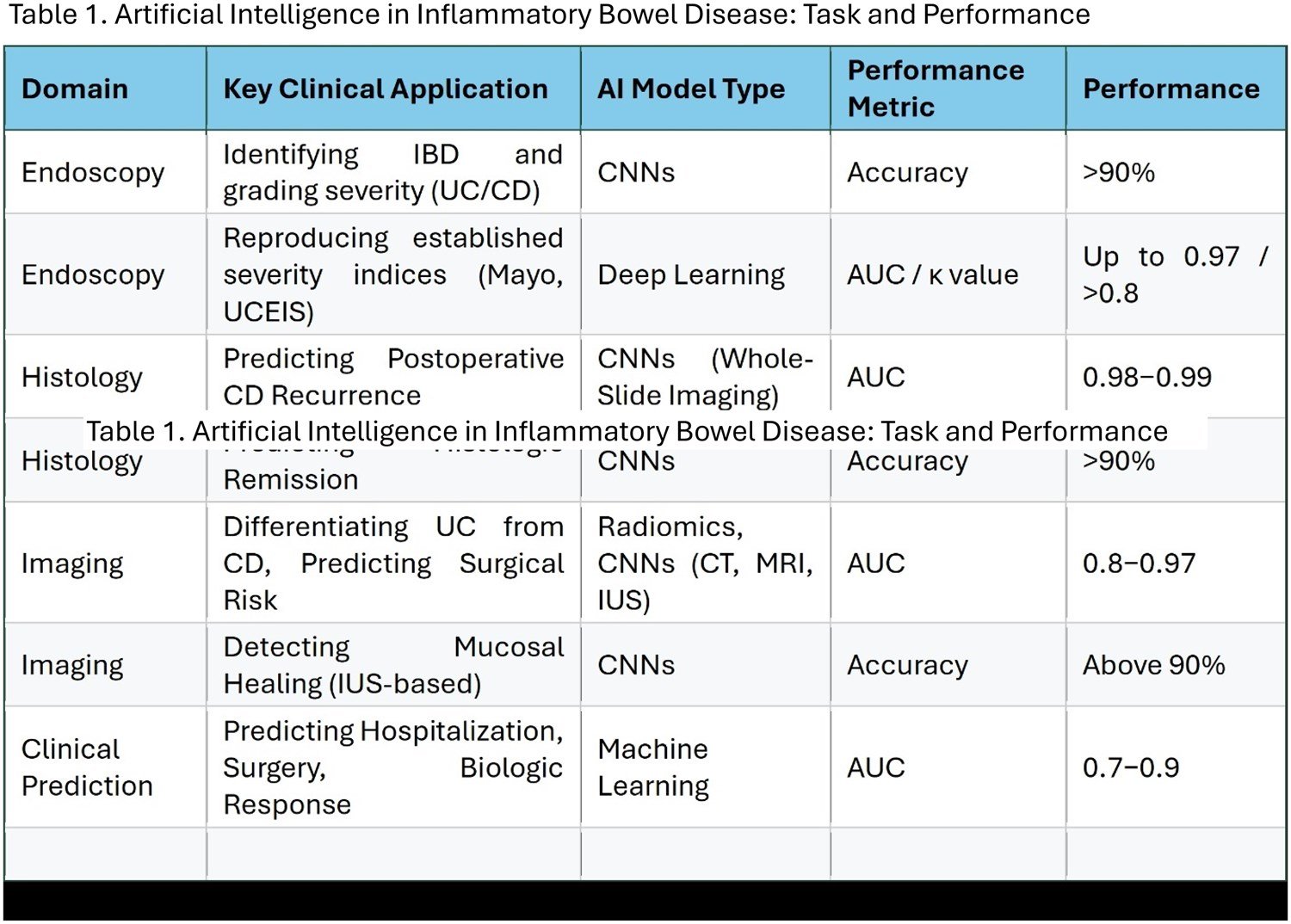

**Funding Agencies:**

None

